# Kinetics and antimicrobial activity of gallic acid by novel bacterial co-culture system using Taguchi’s method and submerged fermentation

**DOI:** 10.1007/s00203-022-03168-2

**Published:** 2022-09-01

**Authors:** Subbalaxmi Selvaraj, Julia Moraes Amaral, Vytla Ramachandra Murty

**Affiliations:** 1 Department of Biotechnology, Manipal Institute of Technology (MIT), Manipal Academy of Higher Education (MAHE), Manipal, 576104 India; 2 School of Pharmaceutical Sciences, Universidade Estadual Paulista, Araraquara, Brazil

**Keywords:** Bacterial co-culture, Gallic acid, Taguchi design, Kinetics, Antimicrobial activity

## Abstract

A tannase-positive *Bacillus gottheilii* M2S2 and *Bacillus cereus* M1GT were co-cultivated for the production of gallic acid using tannic acid as the sole carbon source through submerged fermentation. Taguchi orthogonal array of design of experimental methodology was used to estimate the influence and significance of tannic acid concentration, glucose concentration, agitation speed, and inoculum size on the gallic acid production in a shake flask. Among all the factors, agitation speed contributed the highest for gallic acid production (28.28%), followed by glucose concentration (21.59%), inoculum size (19.6%), tannic acid concentration (19.54%), and pH (11.09%). Validation experiments were executed at the found optimized conditions which resulted in a 6.36-fold increase in gallic acid yield compared to unoptimized conditions. Further, the kinetics of growth, tannic acid degradation, and gallic acid yield were evaluated at the optimized conditions. The kinetic parameters Y _x/s_, Y _p/s_, and Y _p/x_ were determined as 0.292 mg of cells/mg of tannic acid, 22.2 µg of gallic acid/mg of tannic acid, and 70.76 µg of gallic acid/mg of cells with a growth rate of 0.273 h ^−1^ after 24 h of fermentation. Finally, the antimicrobial activity of the product gallic acid was investigated against food-borne pathogenic *E. coli, S. aureus, and Serriatia marcescens* and showed a zone of inhibition of 2 cm, 1.6 cm, and 1.3 cm, respectively, using the agar disc diffusion technique. Thus, the cost-effective bioproduct gallic acid proved to be potentially effective to control food poisoning diseases and preserve foodstuff.

**Supplementary Information:**

The online version contains supplementary material available at 10.1007/s00203-022-03168-2.

## Introduction

3,4,5-trihydroxy benzoic acid is an organic compound commonly known as Gallic acid, discovered by Carl Wilhelm Scheele in 1878. It is an integral compound of tannic acid molecules found in plants and fruits (Aguilar-Zarate et al. 2015; Chandrasekaran and Beena, 2013). Gallic acid devises wide applications in the synthesis of an antibacterial drug called trimethoprim (pharmaceutical industry); in the synthesis of pyrogallol, inks, dihydroxyacetone, alkaloids, and photographic developer (chemical industry); and in the production of an antioxidant, propyl gallate, and gallate esters, as preservatives (food industry) (Kanpiengjai et al. 2020; Banerjee et al. 2007). It is also known to exhibit several pharmacological properties like antibacterial, antiallergic, antioxidant, antimutagenic, anti-inflammatory, neuroprotective, and anticarcinogenic activities (Kanpiengjai et al. 2020; Verma et al. 2013; Bajpai and Patil 2008; Kroes et al. 1992). Gallic acid being a phenolic component showed delayed antimicrobial resistance against pathogens of bovine respiratory diseases (Rajamanickam et al. 2019). Gallic acid also causes damage to the sensitive strains by penetrating the cell membranes and chelating with the calcium ions which results in the death of pathogens (Sarjit et al., 2015), which represents that gallic acid is a potent antimicrobial product. The global yearly demand for gallic acid is about 8000 tonnes, 75% of it is used in the production of trimethoprim (Chandrasekaran and Beena, 2013; Lokeswari, 2010a), also the natural existence is limited.

In India, gallic acid is manufactured by extracting tannic acid from imported tara powder and acid hydrolyzing it with sulfuric acid at high temperatures to obtain gallic acid which is crystallized to obtain the final product trimethoprim (an antibacterial agent). The manufacture of trimethoprim involves three stages, viz;

$$Tara powder--Stage I------\to Gallic acid$$

$$Gallic acid-----Stage II----\to \mathrm{3,4},5-Trimethoxy benzaldehyde$$

$$\mathrm{3,4},5-Trimethoxy benzaldehyde----Stage III-----\to Trimethoprim$$

While most of the manufacturers of trimethoprim are having the technology of stage III, stages I and II are not very much established. Indian companies have, by and large, been successful in achieving good efficiencies. Even though the production process is somewhat simple and their production units have been stabilized, the important raw material tara powder is expensive as it is presently imported and also has irregular supply demanding an alternate source. The major constituent of tara powder is tannic acid about 40%. Conventionally, the compound 3,4,5 trimethoxy benzaldehyde is produced by chemical methods, but these processes generate not only the environment but also processing problems like low purity, high cost, and low yield. Therefore, this gallic acid for the synthesis of drug intermediates stands unsuitable. On the other hand, microbial tannase can be used to hydrolysis tannins into gallic acid which is a simpler method, requires fewer stages, and is a non-polluting process (Chandrasekaran and Beena, 2013; Lokeswari 2010a; Lokeswari et al. 2010b). The tannase is an inducible enzyme secreted by microbes, plants, and animals, upon hydrolysis of tannic acid, liberates gallic acid and glucose in the ratio of 9:1. Among them, microbial tannase mainly from fungi is well reported (Arshad et al. 2019; Cruz et al. 2017; Xiao et al. 2015; Mondal et al. 2001). Still, the documents on the degradation of tannins are scarce. Predominantly used strain for the hydrolysis of tannic acid into gallic acid was *Aspergilli* (Saeed et al. 2020; Arshad et al. 2019; Lokeswari 2010a; Bajpai and Patil, 2008; Banerjee et al. 2007), among bacteria *Enterobacter* sp. (Sharma et al. 2017), *Bacillus subtilis* AM1 (Aguilar-Zarate et al. 2015), *Lactobacillus plantarum* CIR1 (Aguilar-Zarate et al. 2015), *Corynebacterium* sp. (Deschamps and Lebeault, 1984), *Klebsiella pneumonia* (Deschamps and Lebeault, 1984), etc. have been reported to yield gallic acid.

The gallic acid and tannase can be co-produced using both submerged fermentation (SmF) and solid state fermentation (SSF) processes (Belur and Mugeraya, 2011). Yet, submerged fermentation is the most ideal choice for bacterial growth than SSF as they need high moisture content, doubling time is short, purification of the product is easier, and control of the fermentation is simpler (Belur and Mugeraya, 2011). Upon the unearthing of an enzyme tannase in 1867, excessive research was carried out for its products mainly from fungus, as tannins were considered bacteriostatic. In the year 1983, the very first finding of bacterial tannase was reported (Deschamps et al. 1983) and the strong focus on bacterial tannase and gallic acid synthesis was seen from 1990 onwards (Mondal et al. 2001; Das Mohapatra et al. 2006; Selwal et al. 2010; Natarajan and Rajendran, 2012). In a natural habitat, fermentations generally occur in mixed cultures. The mixed culture may show interactions among themselves and exhibit kinetic reactions (Purohit et al. 2006). These interactions of the microbial population also depend on the environmental factors and create two types of association, synergistic (proliferates the microorganisms and increases the metabolite or enzyme production) and antagonistic (inhibits the growth of other microorganisms) (Abdullah et al. 2018). Bacterial co-culturing is a unique method in which two or more different cultures develop under the same environmental conditions with some synergy among them. In this study, two different bacterial strains with a positive synergy were used to improve the metabolite gallic acid and enzyme tannase synthesis rather than bacterial monoculture. There are quite a few articles reported on bacterial co-culture to enhance enzyme productions like alpha-amylase (Abdullah et al. 2018; Pandey et al. 1999). Though, this is the first report on the production of gallic acid from bacterial strains using the co-culturing method.

In this communication, we made attempts to enhance gallic acid production by co-culturing *Bacillus gottheilii* M2S2 and *Bacillus cereus* M1GT through SmF. Taguchi orthogonal array of design of experimental technique was used to study the effects of process parameters, including tannic acid concentration, glucose concentration, agitation speed, inoculum size, and initial pH. Besides, to understand the design of fermentation processes utilizing the co-culturing method, the kinetic constants µ (specific growth rate), X_m_ (maximum biomass concentration), Y_x/s_ (biomass yield coefficient based on substrate consumption), Y_p/s_ (product yield coefficient based on substrate consumption), and Y_p/x_ (product yield coefficient based on biomass) were evaluated at the optimized process conditions. Finally, the product gallic acid produced was used to test the antimicrobial activity against the bacterial strains causing food poisoning diseases.

## Materials and methods

### Microorganism and bacterial co-culture cell suspension preparation

Gallic acid-producing strains of *Bacillus gottheilii* M2S2 and *Bacillus cereus* M1GT were previously isolated from tannery effluent soil and gastrointestinal tract of goat, respectively, by adopting liquid enrichment and spread plate methods as described elsewhere (Subbalaxmi and Murty, 2016; Selvaraj et al. 2021). The ribosomal RNA gene sequence of newly isolated *Bacillus gottheilii* M2S2 and *Bacillus cereus* M1GT was deposited at Gen Bank bearing an accession ID as KU866380 and KX033490, respectively.

A one loop-full of strain *Bacillus gottheilii* M2S2 or *Bacillus cereus* M1GT was grown in 10 mL of sterilized nutrient broth for 20 h at 180 rpm and 32 °C. Then, 1 mL of the respective strain was further grown in 50 mL sterilized nutrient broth under the same condition. Further to obtain co-culture suspension, the individual suspensions of *Bacillus gottheilii* M2S2 and *Bacillus cereus* M1GT were mixed in equal proportions of volume ratio of 1:1 as these strains exhibited approximately equal proportions of cell quantity based on the viable cell count experiment (Li et al. 2018).

### Co-culture fermentation

To run the co-culture submerged fermentation, 2 mL of 20 h old *Bacillus gottheilii* M2S2 inoculum and 2 mL of 20 h old *Bacillus cereus* M1GT inoculum each containing 4 × 10^11^ CFU/mL were aseptically mixed into 250 mL Erlenmeyer flask containing 100 mL of production media composed of 1% (w/v) of tannic acid (as carbon source and tannase inducer), 0.5% (w/v) of NH_4_NO_3_ (as nitrogen source), and supplemented with 0.05% (w/v) of K_2_HPO_4_, 0.05% (w/v) of KH_2_PO_4_, 0.05% (w/v) of MgSO_4_.7H_2_O, 0.05% (w/v) of NaCl; adjusted the pH to 5.0. The Erlenmeyer flasks were incubated in a rotary shaker at 32 °C and 180 rpm for 24 h (Purohit et al. 2006; Banerjee et al. 2005). Experiments were carried out in triplicates and after fermentation, the gallic acid content, tannic acid content, biomass, and pH were determined.

### Optimization of gallic acid production using Taguchi’s L_16_ orthogonal array design

Based on our preliminary studies using co-culture of *Bacillus gottheilii* M2S2 and *Bacillus cereus* M1GT, process parameters such as tannic acid concentration, glucose concentration, agitation speed, initial pH, and inoculum size were found to be the most influential parameters for gallic acid production. Since the variations in these parameters can alter the course of an experiment, in turn, the “response parameter”, i.e., gallic acid concentration can be increased, thereby helping to set the experimental parameters while designing an experiment.

To study the effect of both chemical and physical parameters on gallic acid production by bacterial co-culture and to enhance its concentration for maximum gallic acid production an ordered L_16_ orthogonal array of experiments was used. The above-mentioned five parameters were evaluated and optimized at four different levels designated by L1, L2, L3, and L4 as with 16 experimental runs as depicted in Table 1. Concerning the optimization of process conditions, each column in Table 1 would represent specific process parameters, and each row would depict experimental runs with different combinations of parameters. The main aim of the optimization of process conditions was to enhance gallic acid production. For this, Taguchi’s statistical method was used to find the optimal conditions which are based on the signal-to-noise ratio (S/N) function. This method eliminates the experimental variations improving the overall outcome of the experiment. This is done by setting the ‘signal-to-noise’ ratio as *“larger the better”* (Singh and Verma, 2019; Mohan and Reddy, 2013). The main aim of this statistical method is to find the optimal experimental conditions to enhance the yield of gallic acid by using the aforementioned option. Here, the gallic acid concentration is measured as a response, and the formula for the S/N ratio is given:

1 $$\frac{S}{N}= -10\,log\left[\frac{i}{N} \sum_{N=1}^{N}\left(\frac{1}{{{X}_{i}}^{2}}\right)\right],$$

where S/N = signal-to-noise ratio; *N* = number of experimental runs; and *X*_*i*_ = gallic acid concentration of respective runs. The optimal values of each parameter are those at the largest S/N ratio.

**Table 1:** Taguchi’s L_16_ orthogonal array of experimental design matrix for the production of gallic acid by co-culturing *Bacillus gottheilii* M2S2 and *Bacillus cereus* M1GT under submerged fermentation in a shake flask

Run	A	B	C	D	E	Gallic acid concentration (µg/mL)		S/N ratio
						Experimental	Predicted	
1	0.5 (L1)	0.0 (L1)	100 (L1)	4 (L1)	4 (L1)	279.00 ± 0.88	279.00	48.91
2	0.5 (L1)	0.2 (L2)	120 (L2)	5 (L2)	6 (L2)	193.63 ± 1.03	193.63	45.74
3	0.5 (L1)	0.3 (L3)	140 (L3)	6 (L3)	8 (L3)	319.83 ± 0.93	319.83	50.09
4	0.5 (L1)	0.4 (L4)	180 (L4)	7 (L4)	10 (L4)	441.70 ± 0.82	441.70	52.90
5	1.0 (L2)	0.0 (L1)	120 (L2)	6 (L3)	10 (L4)	574.08 ± 0.79	574.08	55.18
6	1.0 (L2)	0.2 (L2)	100 (L1)	7 (L4)	8 (L3)	292.00 ± 0.91	292.00	49.31
7	1.0 (L2)	0.3 (L3)	180 (L4)	4 (L1)	6 (L2)	222.08 ± 1.12	222.08	46.93
8	1.0 (L2)	0.4 (L4)	140 (L3)	5 (L2)	4 (L1)	210.95 ± 0.95	210.95	46.48
9	1.5 (L3)	0.0 (L1)	140 (L3)	7 (L4)	6 (L2)	218.99 ± 0.89	218.99	46.81
10	1.5 (L3)	0.2 (L2)	180 (L4)	6 (L3)	4 (L1)	168.26 ± 1.20	168.26	44.52
11	1.5 (L3)	0.3 (L3)	100 (L1)	5 (L2)	10 (L4)	107.64 ± 1.42	107.64	40.64
12	1.5 (L3)	0.4 (L4)	120 (L2)	4 (L1)	8 (L3)	354.47 ± 0.96	354.47	50.99
13	2.0 (L4)	0.0 (L1)	180 (L4)	5 (L2)	8 (L3)	319.21 ± 0.75	319.21	50.08
14	2.0 (L4)	0.2 (L2)	140 (L3)	4 (L1)	10 (L4)	87.23 ± 1.06	87.23	38.81
15	2.0 (L4)	0.3 (L3)	120 (L2)	7 (L4)	4 (L1)	311.78 ± 0.69	311.78	49.88
16	2.0 (L4)	0.4 (L4)	100 (L1)	6 (L3)	6 (L2)	79.18 ± 0.98	79.18	37.97

L1, L2, L3, and L4 are the four different levels of parameters

*A* tannic acid concentration (% w/v), *B* glucose concentration (% w/v), *C* agitation speed (rpm), *D* initial pH, *E* inoculum size (% v/v)

The design matrix, its analysis, and the process of experimental runs were evaluated using statistical software MINITAB 17 (Trial version). The evaluated data were taken to rank the most influential parameters on the yield of gallic acid and to find the best fermentation environments for co-culture of *Bacillus gottheilii* M2S2 and *Bacillus cereus* M1GT to produce the highest gallic acid. To validate Taguchi’s L_16_ orthogonal array design, the experiments were carried out in triplicates at determining optimized process conditions.

### Kinetic studies of gallic acid production at optimized process conditions

The kinetics of gallic acid production from co-culture of *Bacillus gottheilii* M2S2 and *Bacillus cereus* M1GT was evaluated in optimized conditions, which was found based on the Taguchi’s L_16_ orthogonal array approach as described below. The composition of fermentation media and cultural conditions are as follows: tannic acid 1% (w/v), agitation speed 120 rpm, and inoculum size 10% (v/v); whereas other components were maintained at 0.5% (w/v) of NH_4_NO_3_, 0.05% (w/v) of K_2_HPO_4_, 0.05% (w/v) of KH_2_PO_4_, 0.05% (w/v) of MgSO_4_.7H_2_O, and 0.05% (w/v) of NaCl; at optimum pH 6.0. Then, for every 4 h of fermentation, tannic acid content, gallic acid, biomass concentration, total protein, and glucose were determined. Experiments were carried out independently in triplicates.

To better understand the bacterial co-culture of gallic acid production, different kinetic parameters, i.e., specific growth rate (µ), biomass yield coefficient based on substrate utilization (Y_xs_), product yield coefficient based on biomass (Y_px_), product yield coefficient based on substrate utilization (Y_ps_), the specific rate of substrate utilization (q_s_), and specific rate of product formation (q_p_) were estimated based on the methods described by Doran (1995). The following are the set of equations used to estimate the fermentation kinetic parameters:

2 $$\mu = \frac{1}{X}\frac{dX}{dt},$$

3 $${Y}_{xs}= \frac{dX/dt}{dS/dt},$$

4 $${q}_{s}= \frac{1}{X}dS/dt,$$

5 $${Y}_{ps}= \frac{dP/dt}{ds/dt},$$

6 $${Y}_{px}= \frac{dP/dt}{dX/dt},$$

7 $${q}_{p}= \frac{1}{X}dP/dt.$$

In the above equations, *µ*, *X*, *S*, *P*, and *t* are specific growth rate, biomass concentration, substrate (tannic acid) concentration, product (gallic acid) concentration, and the fermentation time, respectively. All these fermentation kinetic parameters were studied in the batch process mode at optimized environmental conditions.

### Analytical methods

After submerged fermentation, the total fermentation broth was subjected to centrifugation (10,000 rpm, 15 min, and 4 °C). The cell-free extract was utilized for analysis of gallic acid, tannic acid, and final pH; whereas wet residue (pellet) was used to determine dry cell weight.

The product gallic acid was estimated spectrophotometrically using rhodanine as a coloring agent and methyl gallate as a substrate (Sharma et al. 2000). The amount of gallic acid released was correlated with the gallic acid standard curve, which was attained by measuring the absorbance of different concentrations of standard gallic acid solutions ranging from 0 to 100 nmol.

Further, the protein precipitation method of Ann-Hagerman and Larry-Butler (1978) was adopted to quantify the tannic acid content in the cell-free extract.

Finally, the dry cell weight method was used to determine the biomass concentration; the pellet obtained after centrifugation of fermentation broth was dried at 80 °C in a hot air oven until it reaches constant weight. The biomass concentration was calculated as defined below:

8 $$Biomass\,concentration\left(\frac{g}{L}\right)= \frac{\left(weight\,of\,centrifuge\,tube\,with\,pellet\right)-\left(weight\,of\,empty\,centrifuge\right)}{Volume\,taken\,for\,centrifugation}.$$

### Antibacterial activity of the gallic acid

#### Bacterial strains

The effectiveness of antibacterial activity of gallic acid produced by co-culture of *Bacillus gottheilii* M2S2 and *Bacillus cereus* M1GT under SmF was estimated using two strains of Gram-positive (*Bacillus* species and *Staphylococcus*) and two strains of Gram-negative (*Escherichia coli and Serratia marcescens*) bacteria causing food poisoning disease. The above-mentioned strains for antibacterial check are procured from the culture collection of the Institute of Microbial Technology, Chandigarh, India.

#### Inoculum preparations

The above-mentioned bacterial strains were grown overnight in Nutrient agar slants at 32 °C. Further, the growth of bacterial strains was harvested and diluted using sterile saline water to obtain a viable cell count of 10^6^ CFU/mL and its absorbance was adjusted at 600 nm using a spectrophotometer.

#### Antibacterial activity of gallic acid produced

The diffusion assay method was adopted to estimate the antimicrobial activity of the gallic acid produced under SmF by co-culture fermentation of *Bacillus gottheilii* M2S2 and *Bacillus cereus* M1GT. The gallic acid extract was steam-sterilized at 121 °C and 15 psi for 15 min. The overnight grown bacterial suspension of 0.1 mL was spread plated on petri dishes containing 20 mL solidified nutrient agar medium. Further, the sterilized cork borer holes were punched on nutrient agar plates. Then, the gallic acid solution of 0.5 mL was poured into the well and the plates were kept in the incubator for 24 h at 37 °C. The exhibition of clear zones was recorded by the Vernier caliper and considered as the presence of antibacterial activity.

## Results and discussion

Microbial production of metabolites mainly depends on the physical and chemical environment of the microorganism employed. Initially, the production of gallic acid was studied by co-culture fermentation of *Bacillus gottheilii* M2S2 and *Bacillus cereus* M1GT at 32 °C, 180 rpm and for 48 h in production medium (% w/v) comprising tannic acid, 1; NH_4_NO_3_, 0.5; K_2_HPO_4_, 0.05; KH_2_PO4, 0.05; MgSO4. 7H_2_O, 0.05; and NaCl, 0.05; with pH of 5.0. The maximum gallic acid concentration of 22.76 µg/mL was exhibited at 24 h of fermentation. The product gallic acid was found to be growth associated and also similar results have been observed in *Lactobacillus plantarum* (Natarajan et al. 2011) and *Bacillus sphericus* (Raghuwanshi et al. 2011). Based on the preliminary studies, the parameters such as tannic acid (carbon source), glucose (inducer), inoculum size, initial pH, and agitation speed were found to be most critical for the production of gallic acid by co-culture fermentation of *Bacillus gottheilii* M2S2 and *Bacillus cereus* M1GT.

Yet, the interaction effects of these parameters on the gallic acid yield were not possible with traditional methods of optimization, which has proven to exhibit a significant effect on the regulation of metabolism (Subbalaxmi and Murty, 2016). Hence, optimization of fermentation media was examined for gallic acid yield with five critical parameters at their selected levels as shown in Table 1.

### Regression analysis

The co-culturing of *Bacillus gottheilii* M2S2 and *Bacillus cereus* M1GT was adopted in this study and the strains have shown a good yield of gallic acid in the initial study completed. As the bacterial co-culture production of gallic acid was not reported, hence this study aimed to check the prospect of the bacterial cultures producing a high amount of gallic acid on a laboratory scale. The process parameters such as tannic acid concentration, glucose concentration, agitation speed, initial pH, and inoculum size were considered in the development of mathematical models. Table 1 illustrates the final gallic acid concentration at different levels of fermentation media compositions. The Taguchi L_16_ design matrix showed significant differences in the gallic acid yield (Table 1). Among all 16 experimental trials, the maximum and minimum gallic acid concentration was achieved in Run 5 (143.52 µg/mL: Tannic acid, 1% w/v; Glucose, 0% w/v; Agitation speed, 120 rpm; pH, 5 and inoculum size, 10% v/v) and Run 16 (19.79 µg/mL: Tannic acid, 2% w/v; Glucose, 0.4% w/v; Agitation speed, 100 rpm; pH, 6 and inoculum size, 6% v/v), respectively, at 32 °C and 24 h of fermentation. The differences in the concentrations of gallic acid with a change in the media composition indicate that the production of fermentation products is the main function of media composition. This statistical method of optimization showed a significant increase in the gallic acid yield from 22.76 to 143.52 µg/mL when compared to the traditional method of media optimization.

**Fig. 1:**
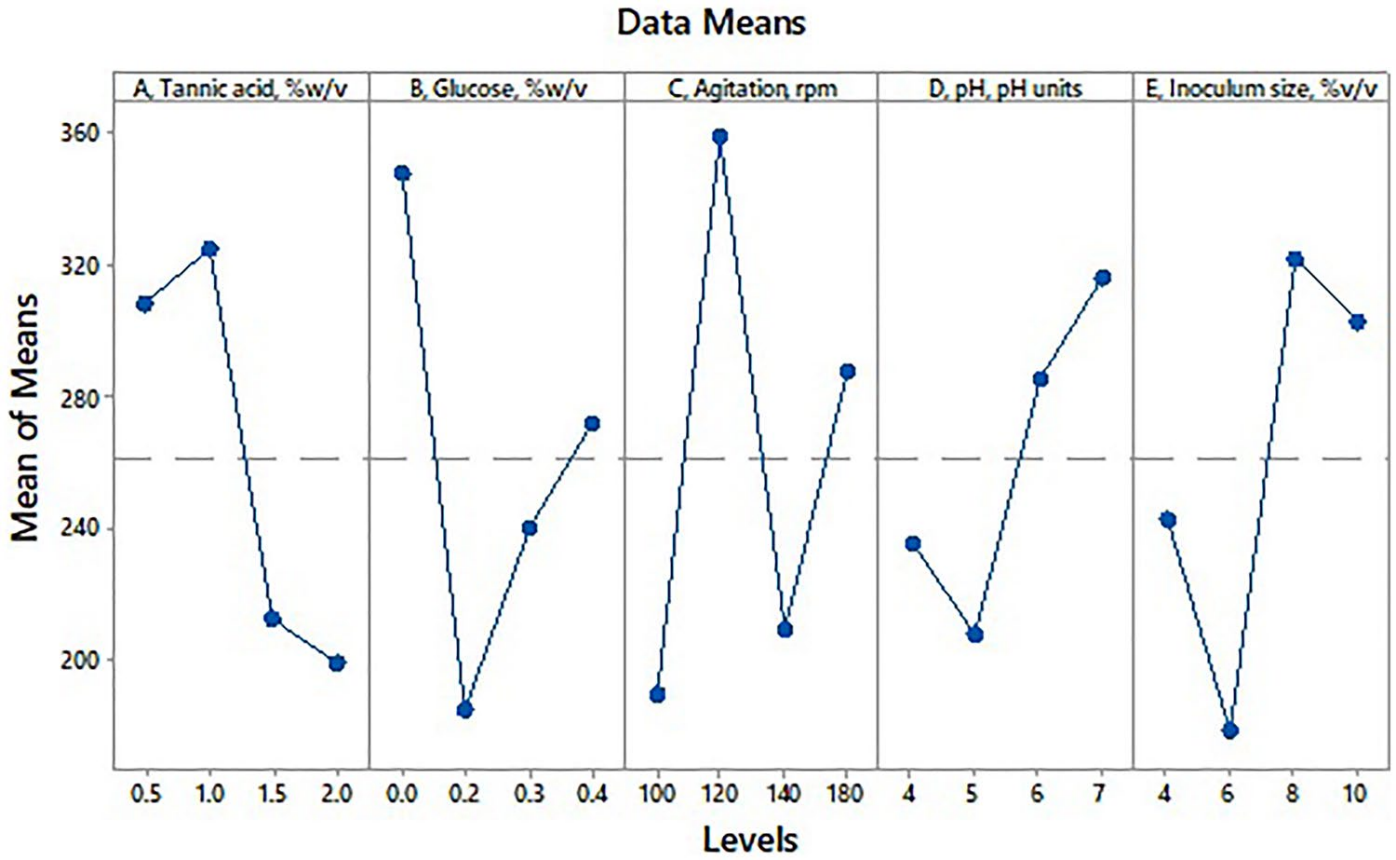
The main effect plots of all individual parameters of tannic acid, glucose, agitation speed, initial pH, and inoculum size on gallic acid yield under SmF by co-culture of *Bacillus gottheilii* M2S2 and *Bacillus cereus* M1GT

The S/N ratio in Taguchi design is generally used to eliminate the experimental variations caused due to uncontrollable parameters. Table 1 depicts the results of the L_16_ orthogonal array for the production of gallic acid, and the S/N ratio (larger is better). It is an important parameter in Taguchi’s L_16_ orthogonal array design to identify the optimal conditions for the process. The values of the S/N ratio tell which combination of parameters has the maximum effect on the response, i.e., gallic acid concentration. The upper value of the S/N ratio indicates that those constituents in composition have the maximum effect on the gallic acid yield. In this study, based on the main effects of each parameter the order of parameters ranked as agitation speed > glucose concentration > inoculum size > tannic acid concentration > initial pH, indicating that agitation speed had the highest effect and initial pH exhibited the least effect on the gallic acid yield by co-culture of *Bacillus gottheilii* M2S2 and *Bacillus cereus* M1GT. The main effect plots of individual parameters are generated by plotting the response average against each parameter level using the software MINITAB (Fig. 1). These plots explain how an individual parameter affects the response, i.e., gallic acid yield. The main effect of a parameter can be negligible or zero if the line is horizontal to the X-axis. If the line makes a larger deviation in vertical location from the horizontal X-axis, then the main effect can be maximum. In this study, it is noticed that tannic acid at level 2 (1% w/v), glucose at level 1 (0% w/v), agitation speed at level 2 (120 rpm), initial pH at level 4 (7.0), and inoculum size at level 3 (8% v/v) exhibited the maximum main effect on the gallic acid yield (Fig. 1). These levels and their values indicate the optimal process conditions for the growth and metabolism of *Bacillus gottheilii* M2S2 and *Bacillus cereus* M1GT. The optimal process conditions can also be predicted from the response table (Table 1).

The response table for the S/N ratio for each parameter and each parameter is ranked based on the delta, which generally relates to the relative degree of the effects of different parameters (Table S1). A parameter with the highest delta is ranked 1. A parameter with a high S/N ratio indicates the better performance of that parameter for the production of gallic acid with the least error of measurement. The graphical representation of the S/N ratio is provided in Figure S1. Agitation speed exhibits the maximum contribution and is ranked 1 for delta followed by inoculum size and concentration of glucose as shown in Table S1. The mean in the Taguchi design represents the average response of gallic acid yield for each combination of parameters and levels. The maximum percent of this contributes greatly to enhancing gallic acid yield. Based on the mean response as shown in Table S1, agitation speed showed maximum influence followed by glucose content, inoculum size, tannic acid concentration, and initial pH.

During the analysis of the effect of each parameter with their levels, the observation was noticed that the highest average effect was seen at the agitation speed of level 2. Subsequently, the next highest were glucose concentration, inoculum size, tannic acid concentration, and initial medium pH at levels 1, 3, 2 and 4, respectively (Fig. 1 and Table S2). The effect of the parameter at the individual level is defined as the difference between the average value at a high and low level of each parameter. Larger the difference, the stronger the significance. The positive or negative signs of values of the effect of different parameters at each level will determine whether the contribution towards gallic acid production has increased or decreased (Table S2 and Table S3). It was found that the relative influence of process parameters on gallic acid is shown below for the fermentation period of 24 h at 32 °C.

Agitation speed > Glucose concentration > Inoculum size > Tannic acid concentration > Initial pH.

### Impact of interaction effects of parameters

The MINITAB 17.0 software generated the interaction effects and was examined individually to better understand the production of gallic acid by co-culture of *Bacillus gottheilii* M2S2 and *Bacillus cereus* M1GT. The severity index (SI) helps to understand the interaction of two parameters on gallic acid yield at defined levels. The Severity Index measures the angle between the two straight interaction lines in an interaction plot. SI also indicates the strength of the presence of interaction. The angle between the interaction lines can vary between 0 and 90 degrees and is expressed on a scale of 0–100 as the SI (Table 2). The results of predicted interactions shown in Table 2 are in good agreement with the interaction plots shown in Figures S2, S3, and S4.

**Table 2:** Interaction effect of selected parameters on gallic acid yield with co-culture fermentation of *Bacillus gottheilii* M2S2 and *Bacillus cereus* M1GT under SmF

Interacting parameter pairs	Column^a^	SI (%)	Col^b^	Opt^c^
pH × Glucose	4 × 2	96.81	7	[3,1]
Tannic acid × Glucose	1 × 2	81.19	3	[2,1]
Tannic acid × Agitation speed	1 × 3	73.00	5	[2,2]
Tannic acid × Inoculum size	1 × 5	66.21	9	[2,4]
Inoculum size × pH	5 × 4	60.47	13	[4,3]
Tannic acid × pH	1 × 4	59.91	6	[2,3]
Inoculum size × Glucose	5 × 2	57.50	10	[4,1]
Agitation speed × pH	3 × 4	37.48	9	[2,3]
Agitation speed × Inoculum size	3 × 5	42.34	12	[2,4]
Glucose × Agitation speed	2 × 3	23.46	6	[1,2]

*SI* interacting severity index (100% for 90° angle between the lines, 0% for parallel lines)

^a^locations of column to which interacting parameters are assigned

^b^shows the column that should be reserved if this interaction effect is to be studied

^C^indicates the parameter levels desirable for the optimum conditions

Based on the statistical results represented in Table 2, it can be noticed that the interaction of initial pH and glucose concentration of the media showed the highest effect of 96.81%. But, it is also intrusive to note that the initial pH is having the least percent factor of 11.09% (Table 3) which showed maximum interaction with the parameter glucose concentration (21.59%). Among all the selected parameters, the interaction between glucose concentration and agitation speed exhibited the least severity index of 23.46%. Hence from the interaction studies, it can be established that the effect of individual parameters on gallic acid is different and also in the grouping is independent of the effect of the individual parameter. This result suggests that the insignificant parameters at their levels can be very much significant when interacting with other parameters to enhance the gallic acid yield.

**Table 3:** ANOVA for the yield of gallic acid with co-culture fermentation of *Bacillus gottheilii* M2S2 and *Bacillus cereus* M1GT

	Parameter	DOF (f)	Sum of squares (S)	Variance (V)	Pure Sum (S)	Percent (P)%
1	Tannic acid concentration	3	49,984	16,661	49,984	19.54
2	Glucose concentration	3	55,240	18,413	55,240	21.59
3	Agitation speed	3	72,079	24,026	72,079	28.18
4	Initial pH	3	28,378	9459	28,378	11.09
5	Inoculum size	3	50,138	16,713	50,138	19.60
Other	Error	0				
Total		15	255,818			

### Analysis of variance

The results of Taguchi’s design matrix were analyzed and evaluated the contribution of each parameter toward the yield of gallic acid by Analysis of variance (ANOVA). Further, the quality of the experimental results was given by F ratio. This is a test statistic used for more than a few independent parameters. The test statistics can be calculated as follows:

9 $$Test \,statistic= \frac{system\,variance}{unsystematic\,variance}.$$

If the sum of squares (SS) measures the variance, then the test statistic can be shown as

10 $$Test \,statistic= \frac{{SS}_{Model}}{{SS}_{Residual}}.$$

And the total variance is calculated as, ${SS}_{Total}= {SS}_{Model}+ {SS}_{Residual,}$where SS_Model_, expected variance; and SS_Residual_, random variance.

F ratio and a Mean sum of squares (MS) can be calculated using the formula shown below:

11 $$\mathrm{Fratio}= \frac{\text{Model\,mean\,sum\,of\,squares } \, ({\mathrm{MS}}_{\mathrm{Model}})}{\mathrm{Model\,mean\,sum\,of\,squares}({\mathrm{MS}}_{\mathrm{Residual}})},$$

12 $$\mathrm{Sum\,of\,squares }\left(\mathrm{SS}\right)= \frac{\mathrm{Sum\,of\,squares } \, (\mathrm{SS})}{\mathrm{Degrees \, of \, freedom } \, (\mathrm{df})}.$$

ANOVA was carried out to evaluate the variation in gallic acid production caused due to each parameter and also to estimate the optimal level value of a parameter for the maximum product formation. The ANOVA exhibited the values of the model sum of squares, mean squares, and Variance as 15,988, 3198, and 1066, respectively (Table 3). The model obtained from ANOVA for gallic acid production showed multiple regression coefficients (R^2^) of 0.998, which indicates that the model can explain a 99.8% variation in the gallic acid yield.

Based on the statistical calculations and predictive analysis, the optimized values of the individual parameter are obtained and depicted in Table 4. The statistical analysis of Taguchi’s L_16_ experimental design data for gallic acid yield revealed that agitation speed contributed the maximum effect of 24.32% and initial pH exhibited the least effect of 6.03% on gallic acid production at optimum conditions.

**Table 4:** Performance and optimum conditions for the yield of gallic acid by co-culture fermentation of *Bacillus gottheilii* M2S2 and *Bacillus cereus* M1GT

Parameters	Levels	Level description	Contribution
Tannic acid concentration (% w/v)	2	1	20.31
Glucose concentration. (% w/v)	1	0	27.67
Agitation speed (rpm)	2	120	31.08
Initial pH	3	6	7.7
Inoculum size (% v/v)	4	10	13.23

Total contribution from all parameters = 100%

Current grand average of performance = 261.25 µg/mL

Expected result at optimum conditions = 574.08 µg/mL

The final optimum media composition (% w/v) for an increased gallic acid yield by co-culture of *Bacillus gottheilii* M2S2 and *Bacillus cereus* M1GT is, tannic acid, 1; glucose, 0; agitation speed, 120 rpm; and inoculum size, 10% (v/v); whereas, other components were maintained (% w/v) at 0.5, NH_4_NO_3_; 0.05, K_2_HPO_4_; 0.05, KH_2_PO_4_; 0.05, MgSO_4_.7H_2_O; 0.05, NaCl; and optimum pH 6.0 (Table 4).

To validate the results of the experimental design matrix, trials were carried out in triplicates under the optimized conditions and observed the gallic acid yield of 578.26 µg/mL which is in good agreement with the MINITAB software predicted value of 574.08 µg/mL. This optimum condition produced 578.26 µg/mL of gallic acid concentration in 24 h of fermentation, whereas 91.04 µg/mL was observed before process optimization and hence the yield was enhanced by 6.35-fold by Taguchi’s L_16_ orthogonal array of experimental design.

### Kinetic studies of gallic acid production at optimized process conditions

**Fig. 2:**
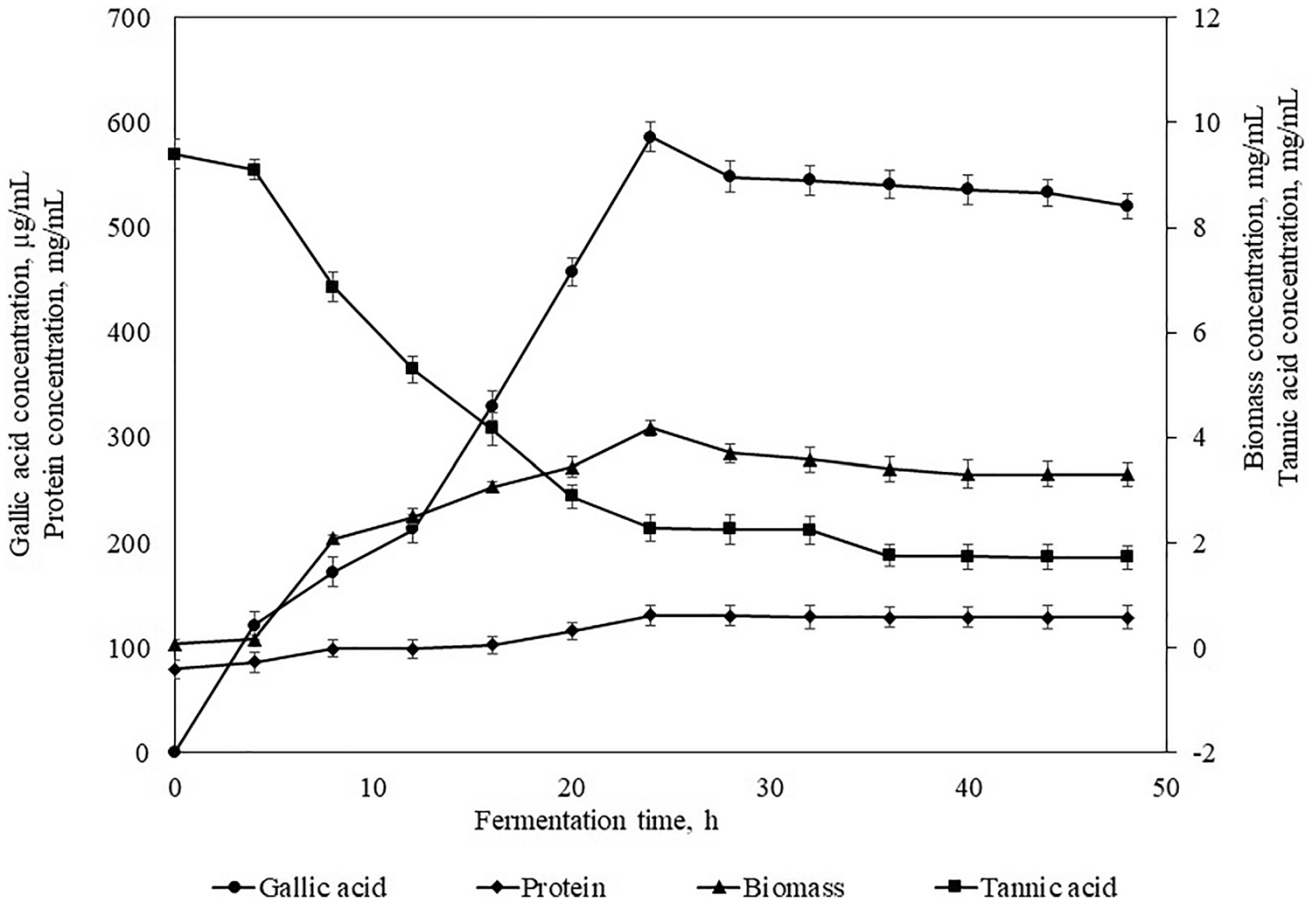
Kinetic profiles of the concentrations of gallic acid, tannic acid, protein, and biomass in the batch process of gallic acid by co-culturing *Bacillus gottheilii* M2S2 and *Bacillus cereus* M1GT at optimized conditions in a shake flask through submerged fermentation

The kinetic results of the co-culture of *Bacillus gottheili* M2S2 and *Bacillus cereus* M1GT in shake flasks for gallic acid yield are shown in Fig. 2. Biomass concentration was inadequate during the initial 4 h of inoculation; this condition is most common during growth where the lag period is vital to acclimatize to the new environment during this period the. During the lag period, the growth was amplified from 0.07 g/L to 0.16 g/L whereas tannin content declined from 9.4 g/L to 9.1 g/L. The lag phase is followed by the log phase, in which the tannic acid content is reduced to 2.28 g/L from 9.1 g/L; whereas, biomass growth was raised to 4.18 g/L from 0.16 g/L. During the log phase, biosynthesis and excretion of gallic acid were initiated, and by the end of the growth phase after 24 h of fermentation, the maximum increase was observed to be 586 µg/mL. Relatively constant biomass and a decrease in gallic acid production were observed in the stationary phase, continuing until the death phase. By these findings, it was demonstrated that the gallic acid yield was growth associated and that both strains of bacteria were rapidly growing strains with tremendous adaptability to the fermentation medium.

During the fermentation process, biomass and product yield factors were estimated (Table 5). After 24 h of fermentation, it was found that maximum Y_p/s_ and Y_p/x_ were 175.95 mg/g of tannic acid and 172.89 mg/g of biomass, respectively. This indicates that gallic acid production followed the growth pattern of *Bacillus gottheilii* M2S2 and *Bacillus cereus* M1GT, indicating that gallic acid was the primary metabolite produced. On further analysis, it was observed 4 h after fermentation that the maximum cell growth rate was 0.701 h^−1^. This demonstrates that both strains grew much faster during the initial exponential phase. For the production of Tannase in SSF under optimized conditions, reported biomass, product yield, and specific growth rate were 0.276 g/g, 0.177 U/g, and 0.0703 h^−1^, respectively (Selvaraj and Vytla, 2017). The evaluation of the specific rate of product formation (q_p_), together with maximum biomass concentration (X_m_) helps in distinguishing whether the increased production rate was due to high biomass production (high X_m_) or because of a very productive strain (high q_p_) (Aguilar et al. 2001). In this study, it was observed that gallic acid production via a co-culture of *Bacillus gottheilii* M2S2 and *Bacillus cereus* M1GT showed more productivity with an average q_p_ of 73.14 µg/mg.h and was also due to the maximum X_m_ of 4.18 mg/mL at 24 h of fermentation (Table 5). The results of this study are in good pact with the outcomes reported by Aguilar et al. (2001). Certain variations in kinetic parameters and growth rate between the previous reports (Selvaraj et al. 2021; Selvaraj and Vytla, 2017) and the current report may have been caused due to differences in fermentation conditions and microbial species. This is the first report which was discussed the impact of kinetic parameters on gallic acid production via the co-culture method.

**Table 5:** Kinetic parameters of growth and gallic acid yield by co-culturing *Bacillus gottheilii* M2S2 and *Bacillus cereus* M1GT at optimized conditions in shake flask through submerged fermentation

Time, h	µ, h^−1^	Y_x/s_ (mg/mg)	q_s_ (mg/mg.h)	Y_p/s_ (µg/mg)	Y_p/x_ (µg/mg)	q_p_ (µg/mg.h)	Gallic acid yield (µg/mL)
0	0	0	0	0	0	0	0
4	0.701	0.1176	24.57	13.32	757.50	320.423	121.20
8	0.423	0.302	1.426	25.05	082.70	34.983	172.02
12	0.298	0.473	0.914	40.27	085.20	36.039	213.00
16	0.236	0.734	0.588	79.15	107.87	45.629	330.08
20	0.194	1.194	0.362	158.94	133.07	56.288	457.76
24	0.166	1.833	0.235	257.017	140.19	59.301	586.00
28	0.140	1.637	0.264	242.478	148.11	62.649	548.00
32	0.123	1.598	0.270	243.286	152.22	64.390	544.96
36	0.109	1.943	0.222	309.04	159.07	67.284	540.82
40	0.098	1.896	0.227	308.21	162.51	68.741	536.28
44	0.089	1.910	0.226	308.22	161.58	68.349	533.22
48	0.082	1.920	0.225	302.43	157.63	66.677	520.18

### Antibacterial activity of gallic acid produced

**Fig. 3:**
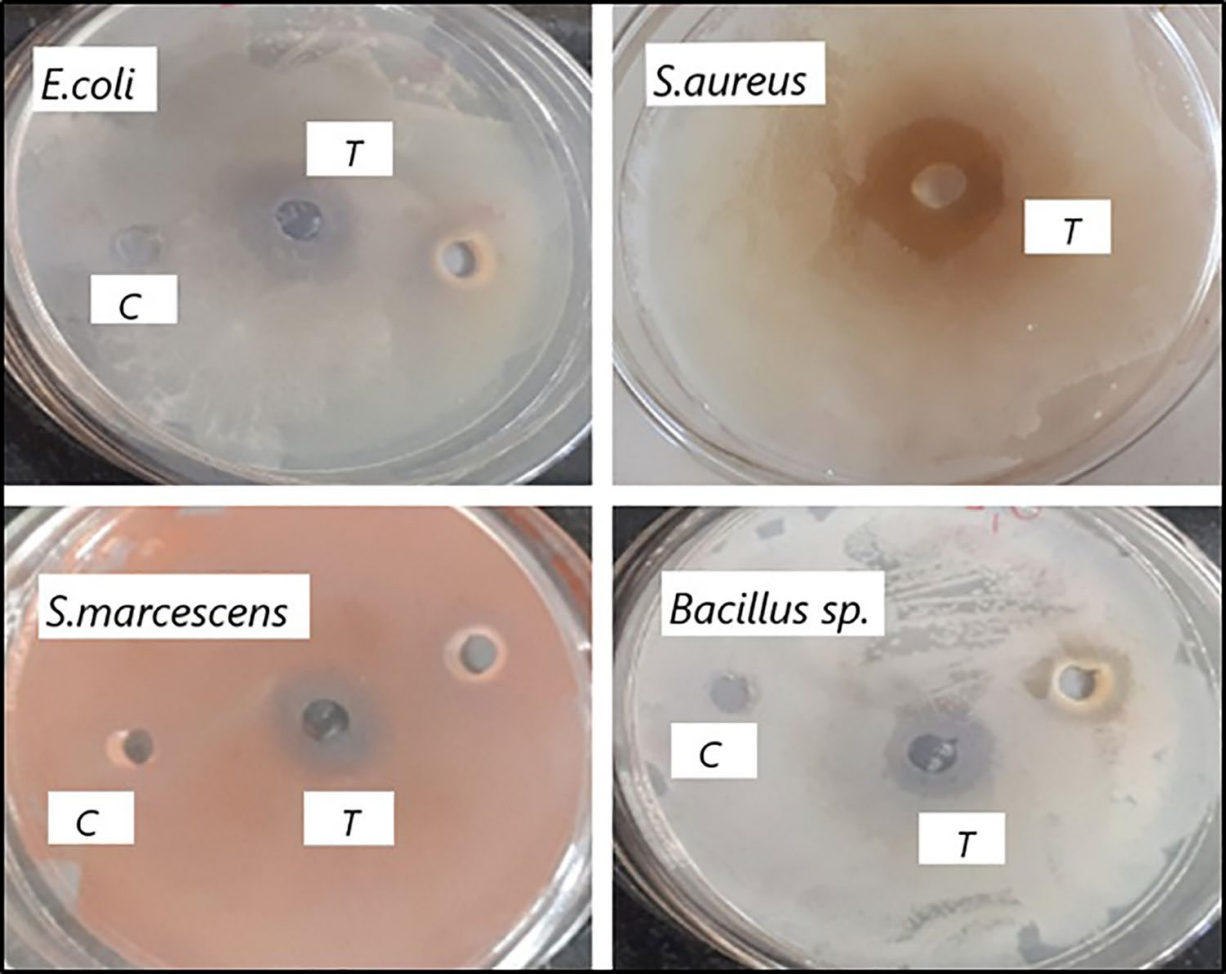
Antimicrobial activity test of the gallic acid extract against food spoilage bacteria. *C* control (without gallic acid extract, i.e., empty well); and *T* test (with gallic acid extract)

In the food industry, the growth of pathogenic strains of bacteria is the main reason for food spoilage. To combat the adverse effects of food spoilage on health, the search for safe, effective, and natural preservatives has been an integral part of the industry. In this present work, the product gallic acid produced through co-culture of *Bacillus gottheilii* M2S2 and *Bacillus cereus* M1GT under submerged fermentation was shown to be natural alternative preservatives for foodstuff and also combats food poisoning, avoiding any health hazards of chemical antimicrobial agent applications. The gallic acid produced under optimized conditions was extracted via cold centrifuge to study the antimicrobial activity. Further investigation of the gallic acid extract was done to estimate the antibacterial activity using the disc diffusion method against four different pathogenic strains of bacteria causing food poisoning—two Gram-positive strains (*Bacillus* sp. and *S. aureus*) and two Gram-negative strains (*E. coli*, *and Serriatia marcescens*). The strains *E. coli*, *S. aureus*, *Serriatia marcescens*, and *Bacillus* sp., showed the ZOI of 20.4 ± 0.38, 16.5 ± 0.41, 13.5 ± 0.43, and 09.4 ± 0.32, respectively, against the product gallic acid. The results of the estimation of antibacterial activity are shown in Fig. 3, and indicate that the gallic acid extract is most prominent in subduing the growth of food spoilage bacteria with variable efficacy. Hence, it can be inferred that the gallic acid extract showed robust antibacterial activity and high effectiveness against pathogenic bacteria. Borges et al. (2013) reported the antimicrobial activity of gallic acid against different pathogenic bacteria like *Escherichia coli*, *Pseudomonas aeruginosa*, *Staphylococcus aureus*, and *Listeria monocytogenes*.

## Conclusion

A maximum gallic acid yield of 574.08 µg/mL was produced after statistical optimization of process conditions, which is a 26- and 27-fold increase when compared to a monoculture of *Bacillus gottheilii* M2S2 and *Bacillus cereus* M1GT, respectively, at unoptimized conditions. The results of kinetic studies indicate that production of gallic acid through co-culture of *Bacillus gottheilii* M2S2 and *Bacillus cereus* M1GT is completely growth associated and 75% of tannic acid was degraded within 24 h of fermentation. A stable balance between growth and yield coefficients was obtained, which indicates that gallic acid production can be scaled up for commercial use. The fed-batch operation can be implemented to maintain the balance between growth and production. The cell-free extract (gallic acid) has proved to be effective against food spoilage pathogens and can be used as an alternative food preservative to store foodstuffs and control food poisoning diseases.

## Supplementary Information

Below is the link to the electronic supplementary material.

Supplementary file1 (DOCX 375 KB)

## Data Availability

The datasets used and/or analyzed during the current study are available from the first author upon reasonable request.
