# Supplementary material for: Kinetics and antimicrobial activity of gallic acid by novel bacterial co-culture system using Taguchi’s method and submerged fermentation
Source: Arch Microbiol. 2022 Sep 1;204(9):584. doi: 10.1007/s00203-022-03168-2 (PMC9436867; doi:10.1007/s00203-022-03168-2)
Supplement: Supplementary file 1 — Supplementary file1 (DOCX 375 KB) [file 203_2022_3168_MOESM1_ESM.docx]

**ARCHIEVES OF MICROBIOLOGY**

**Kinetics and antimicrobial activity of gallic acid by novel bacterial co-culture system using Taguchi’s method and submerged fermentation**

Subbalaxmi Selvaraj^1^, Julia Moraes Amaral^2^, Vytla Ramachandra Murty^1^*

^1^Department of Biotechnology, Manipal Institute of Technology (MIT), Manipal Academy of Higher Education (MAHE), Manipal-576104, India.

^2^School of Pharmaceutical Sciences, Universidade Estadual Paulista, Araraquara-Brazil.

*Corresponding author:

Vytla Ramachandra Murty

Professor

Department of Biotechnology

Manipal Institute of Technology

Manipal-576104, India.

E-mail: murty.vytla@manipal.edu

Fax No.: 91-820-2571071

Phone: +91-9448529691

**Supplementary Materials**

**Table S1** Response table for signal-to-noise ratios.

**Table S2** Response table for Means.

**Table S3** The average effect of parameters at assigned levels for gallic acid production under SmF with co-culture fermentation of *Bacillus gottheilii* M2S2 and *Bacillus cereus* M1GT.

**Figure S1** Main effects plot for SN ratios of gallic acid yield with co-culture fermentation of *Bacillus gottheilii* M2S2 and *Bacillus cereus* M1GT

**Figure S2** Minitab generated interaction plots for the selected factors. (a) D x B; (b) A X B, (c) A X C, and (d) A x E

**Figure S3** Minitab generated interaction plots for the selected factors. (e) E x D; (f) A X D, (g) E X B, and (h) C x D

**Figure S4** Minitab generated interaction plots for the selected factors. (i) C x E; and (j) B X C

Table S1: Response table for signal-to-noise ratios.

| Level | A | B | C | D | E |
| --- | --- | --- | --- | --- | --- |
| 1 | 49.41 | 50.25 | 44.21 | 46.41 | 47.45 |
| 2 | 49.48 | 44.60 | 50.45 | 45.74 | 44.36 |
| 3 | 45.74 | 46.89 | 45.55 | 46.94 | 50.12 |
| 4 | 44.19 | 47.09 | 48.61 | 49.72 | 46.88 |
| Delta | 5.29 | 5.65 | 6.24 | 3.99 | 5.76 |
| Rank | 4 | 3 | 1 | 5 | 2 |
| A, Tannic acid concentration; B, Glucose concentration; C, Agitation speed; D, Initial pH; E, Inoculum size. | | | | | |

Table S2: Response table for Means.

| Level | A | B | C | D | E |
| --- | --- | --- | --- | --- | --- |
| 1 | 308.5 | 347.8 | 189.5 | 235.7 | 242.5 |
| 2 | 324.8 | 185.3 | 358.5 | 207.9 | 178.5 |
| 3 | 212.3 | 240.3 | 209.3 | 285.3 | 321.4 |
| 4 | 199.4 | 271.6 | 287.8 | 316.1 | 302.7 |
| Delta | 125.4 | 162.5 | 169.0 | 108.3 | 142.9 |
| Rank | 4 | 2 | 1 | 5 | 3 |
| A, Tannic acid concentration; B, Glucose concentration; C, Agitation speed; D, Initial pH; E, Inoculum size. | | | | | |

Table S3: The average effect of parameters at assigned levels for gallic acid production under SmF with co-culture fermentation of *Bacillus gottheilii* M2S2 and *Bacillus cereus* M1GT.

| Factors | Level 1 | Level 2 | Level 3 | Level 4 | L2-L1 | L3-L1 | L4-L1 | L3-L2 | L4-L2 | L4-L3 |
| --- | --- | --- | --- | --- | --- | --- | --- | --- | --- | --- |
| A | 308.5 | 324.8 | 212.3 | 199.4 | 16.3 | -96.2 | -109.1 | -112.5 | -125.4 | -12.9 |
| B | 347.8 | 185.3 | 240.3 | 271.6 | -162.5 | -107.5 | -76.2 | 55 | 86.3 | 31.3 |
| C | 189.5 | 358.5 | 209.3 | 287.8 | 169 | 19.8 | 98.3 | -149.2 | -70.7 | 78.5 |
| D | 235.7 | 207.9 | 285.3 | 316.1 | -27.8 | 49.6 | 80.4 | 49.6 | 108.2 | 30.8 |
| E | 242.5 | 178.5 | 321.4 | 302.7 | -64 | 78.9 | 60.2 | 78.9 | 124.2 | -18.7 |
| A, Tannic acid concentration; B, Glucose concentration; C, Agitation speed; D, Initial pH; E, Inoculum size. | | | | | | | | | | |

**2**

**.**

**0**

**1**

**.**

**5**

**1**

**.**

**0**

**0**

**.**

**5**

**5**

**1**

**5**

**0**

**4**

**9**

**4**

**8**

**4**

**7**

**4**

**6**

**4**

**5**

**4**

**4**

**Fig S1** Main effects plot for SN ratios of gallic acid yield with co-culture fermentation of *Bacillus gottheilii* M2S2 and *Bacillus cereus* M1GT


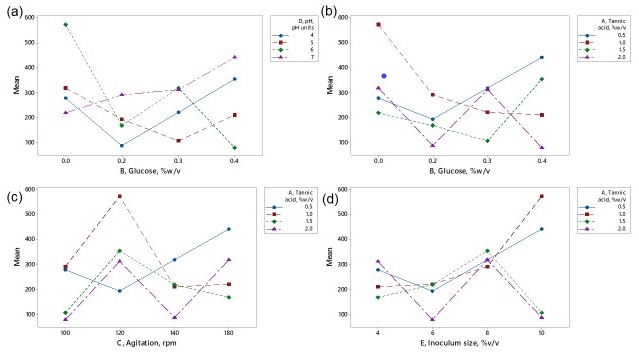
 **Fig S2** Minitab generated interaction plots for the selected factors: (a) D x B; (b) A X B, (c) A X C, and (d) A x E


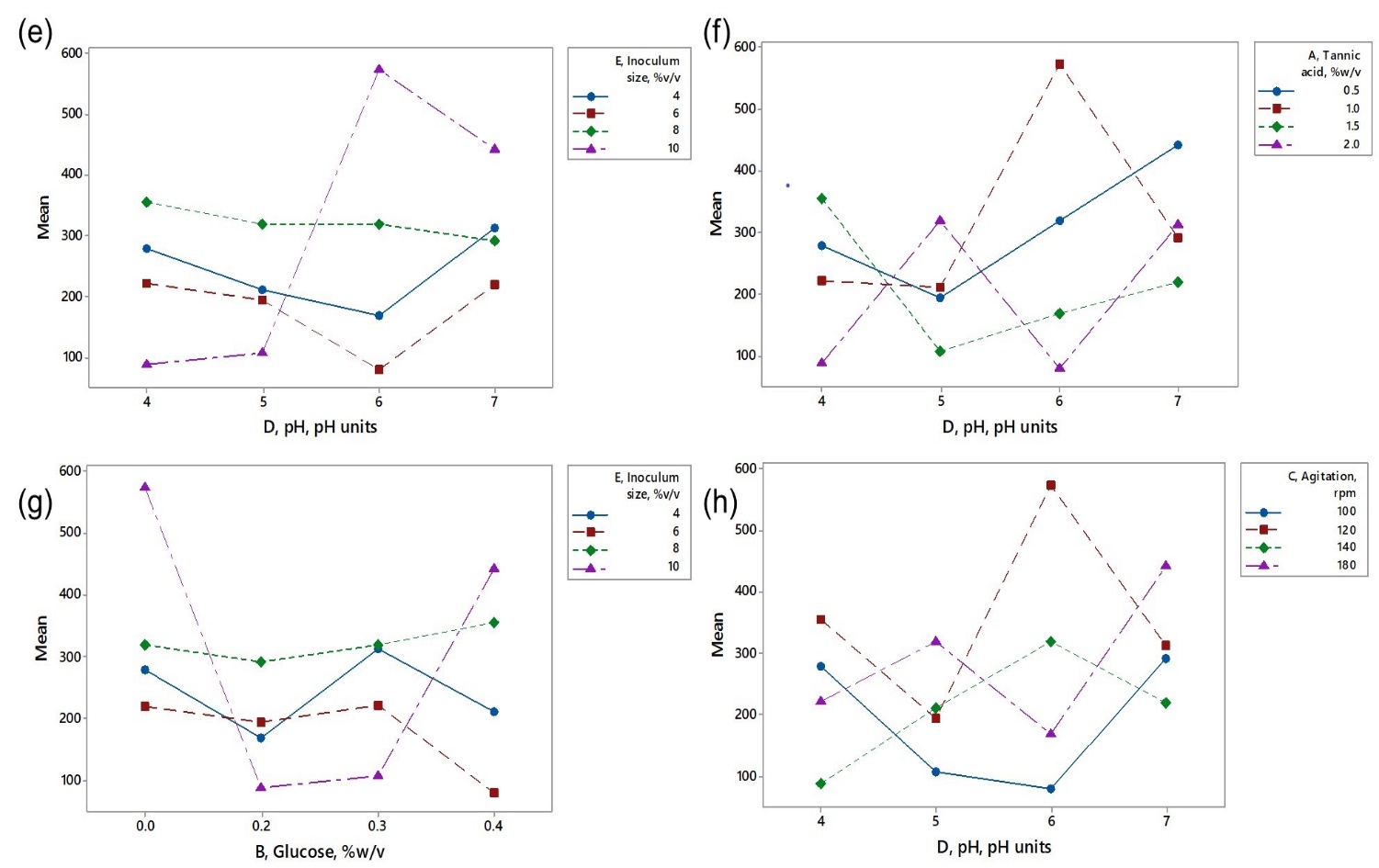
**Fig S3** Minitab generated interaction plots for the selected factors: (e) E x D; (f) A X D, (g) E X B, and (h) C x D


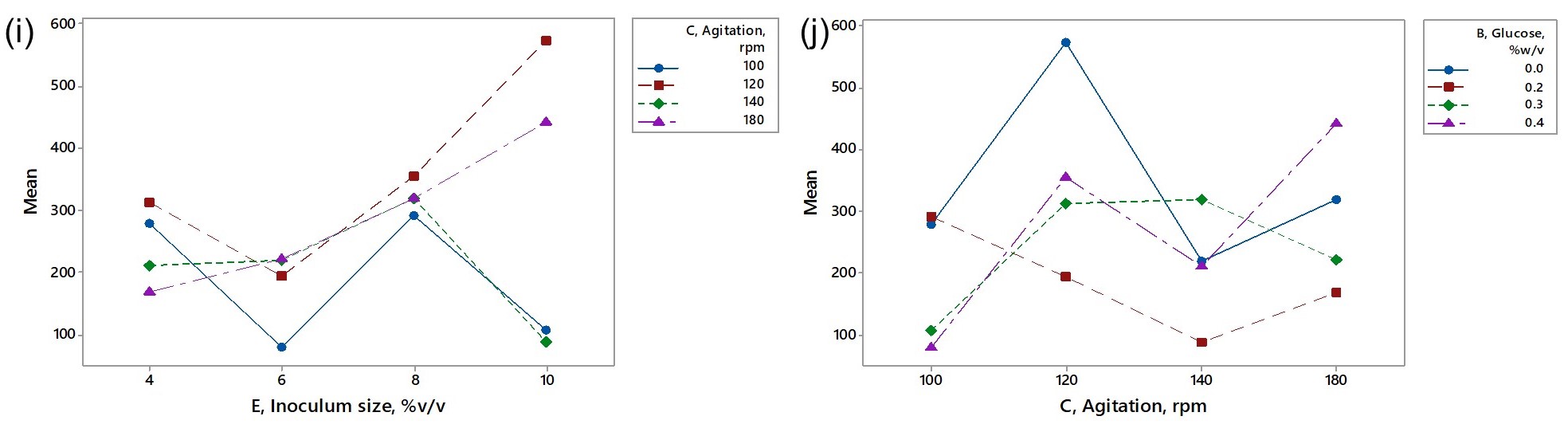
 **Fig S4** Minitab generated interaction plots for the selected factors: (i) C x E; and (j) B X C
